# Evidence-based practices in mental health: Distant dream or emerging reality?

**DOI:** 10.4103/0019-5545.31611

**Published:** 2006

**Authors:** Nimesh G. Desai

In the contemporary scientific environment, practice draws heavily upon evidence. This is particularly so in case of medicine, which has a strong scientific tradition, although the tradition of paternalistic decision-making by experienced senior clinicians has been no less strong. In the traditional model of practice of medicine (including mental health), the science base or the evidence was expected to yield to the wise and considered, even if intuitive, opinion of the treating clinician. This traditional model has been in transformation during the twentieth century, in a variety of ways, and from many directions and forces. Systematic efforts of ‘research to practice’ and expert or consensus guidelines, and clinical practice guidelines (CPGs), have been attempts at translating scientific evidence to clinical practice. The more recent and influential movement of evidence-based medicine (EBM) and evidence-based practice (EBP) takes a decisive position against the traditional model of practice, wherein the individual opinion or wisdom of a clinician is expected to yield to, or at least consider significantly, the available current scientific evidence. Indeed, it moves beyond this reversal to incorporate the principle of medical practice and healthcare being patient-centred, i.e. in terms of individualizing the evidence and encouraging personal choices. It has been said that ‘EBM assumes that patient preferences, expressed as informed choices, always outweigh the scientific evidence’!^1^

The four fundamental principles of EBM have been outlined as (i) use the best available scientific evidence, (ii) individualize the evidence, (iii) incorporate patient preferences, and (iv) expand clinical expertise. The traditional model did not and does not equip the clinicians with adequate expertise to be able to follow these principles and the need to expand the clinical expertise to a wide variety of skills required has also been emphasized.^1^

EBM is about the use of the four fundamental principles to individual treatment decisions in each case. EBPs refer to the forms of healthcare interventions, and the capacity to provide interventions that have been shown to be effective. In other words, EBP is about the extent to which individual practitioners, teams and departments/hospitals, follow and practice the principles of EBM. Further, evidence-based healthcare (EBHC) is about the optimal availability of national/provincial healthcare systems and thus refers to macro policy decisions for creating healthcare systems which are governed by the principles of EBM.

The application of the four fundamental principles are required to guide the decision-making at various levels by individual clinicians/teams in each case for EBM, by departments/institutions as a standard practice for EBP, and by health systems of state or country for EBHC. In a short time, the movement has found wide acceptance and stability in medicine and healthcare. The Institute of Medicine's report^2^ gives the summative description of EBP as ‘the integration of their research evidence, with clinical expertise and patient values’, which is far too basic to be refuted or not be recognized as the way forward. The potential for application of this movement in the field of mental health, especially in developing countries, is worth examining. Is it a distant dream or an emerging reality?

The principle of using the best available scientific evidence is not entirely new to the field of mental health. Parallel to, and sometimes independently ahead of medicine, the field of mental health has generated and documented ‘evidence’ through controlled studies, including the gold standard evidence of randomized controlled trials (RCTs). Such studies have been possible only with well-defined clinical conditions, and not for a wide variety of mental health problems. In the hierarchy of ‘evidence’ in EBM, below the gold standard of RCTs, in that order, are quasi-experimental studies, open clinical trials, systematic observations and unsystematic observations. A large part of the current practice in clinical psychiatry or mental health is based on the type of evidence considered lower in the hierarchy of EBM. This is particularly so for psychological and psychosocial therapies, except for a few elegant studies in those treatment modalities. On the other hand, there are treatment methods with proven efficacy, which do not seem to have reached clinical practice. The task of bridging the gap from ‘research to practice’ was enormous, and seems to have enlarged itself to EBM by incorporating the issues of patient preference and participation.

The task of choosing the best available evidence, and making it easily accessible to clinicians has been attempted by many groups, including the Cochrane collaboration, Schizophrenia Patient Outcome Research Team and Texas Medication Algorithm Project. The task of finding and evaluating the evidence is not easy at all, and the common errors are of confusing CPGs as evidence, or believing that continuing medical education (CME) programmes or accreditation based on CME credits can help clinicians in evaluating evidence. In the more rigorous field of medicine, these myths have been exploded. It is well established and seen in the larger, more vigorous field of medicine that the CPGs developed by professional societies are unsatisfactory, and are often developed to justify their members' practices,^3^ and that attending CME programmes do not change clinician behaviour.^4^ The difficulties for psychiatry and mental health seem to be more due to the constraints of time and pressures of seeing more patients even in the American settings.^5^ Moreover, key element in access to and appropriate use of evidence being the use of computers, it is worth noting that even in countries such as the USA and UK, psychiatry has been noted to ‘remain slow to adopt to information technology’,^6^ and psychiatrists have been found to be individually lagging behind other physicians in the use of information technology, statistically significantly,^7^,^8^ except for younger psychiatrists who use computers at significantly higher rates than their elders.^8^

The relevance of RCT as the gold standard has also been debated. Although it has been argued that in contemporary science, RCTs would form the highest level of evidence, there are concerns of how the evidence may be influenced by the funding support for such studies by the pharmaceutical industry. In a systematic review of RCTs published between 1966 and 2002, it was concluded that ‘systematic bias favours products which are made by the company funding the research. Explanations include the selection of an inappropriate comparator to the product being investigated, and publication bias.’^9^ The recent surge in the market for psychopharmacology products can only compound the phenomenon further.

The second fundamental principle of individualizing the evidence involves the limits of generalizability of evidence to each patient depending on clinical issues of the subtype of the disorder or the comorbidities; as well as of background variables from the age, gender, socioeconomic status, urban domicile to the culture and subculture. It is fascinating to consider how the principle of individualizing evidence to each patient will be applied to the multi-axial system of classifications.

The third fundamental principle of incorporating patient preferences has been a result of the patient-centred medicine/healthcare, and the consumer movement in healthcare, and makes a process of shared decision-making essential. This process, which encourages patients to take more responsibility for making informed choices and adhering to the treatment, makes many clinicians distinctly uncomfortable, if not apprehensive, particularly in mental health.^10^ Difficulties encountered in assessing shared decision-making with mentally ill persons cannot be brushed aside; at the same time, it is worth considering whether traditional values and belief systems of clinicians are likely to hamper the possibility of meaningful partnership with consumers of mental health services. The reportedly successful endeavour, by the National Alliance of Mentally Ill (NAMI), in the USA with service providers and health systems does raise hopes.^11^ The experiences of NAMI are described more for EBP than for EBM wherein specific individual patient/person-related decisions need to be made. Since the EBM clearly would not permit the use of treatment methods not considered suitable/beneficial by a patient for oneself, if follows that the right to refuse treatment converges with the EBM movement. The sociocultural context of shared decision-making process in the Afro-Asian countries will have to be studied and operationalized.

The fourth fundamental principle of expanding clinical expertise is crucial since it refers to the need for equipping individual clinicians and health systems to be able to effectively deliver the services based on the first three principles. Traditional skills of individual clinicians and clinical teams need to be diversified and enlarged considerably to include the analysis of scientific evidence, apply that to individual's sociocultural context, and lead the entire process of dialogue with the patient and the family with effective bilateral communication and interpersonal skills.^12^ Incorporating the principles of EBM and the necessary skills in the curriculum of new professionals being trained is far simpler than altering the perceptions and functioning styles of those previously trained. It has been attempted with modest success in the USA, but the challenge lies in developing countries, specifically in mental health services. Inadequate access/utilization of information technology, together with aggressive marketing and promotion by the pharmaceutical industry (international and national), make the perception of EBM to be a distant dream.

On the other hand, the evolving social, economic and legal trends in developing countries, which are similar to the trends in developed countries, are likely to make the movement for EBM and EBP unavoidable and inescapable. The scientific feasibility and the ethical correctness of the movement needs to be recognized as an emerging reality. It would seem that the need for a genuine attempt at all levels for moving towards the implementation of the principles of EBP, to the extent feasible, is a sociopolitical imperative. Indeed, the seemingly distant dream of EBM, EBP and EBHC can be meaningfully synthesized with the emerging reality of this imperative, so that the dream is fulfilled! The EBP orientation and a pervasive liberal flavouring of mental healthcare services with the principles of EBP can be the first step towards making the dream into a reality. The field of mental health in the developing world would do well to be able to start with this effectively. The attitudinal paradigm shifts and modification of behaviour patterns required across service providers, policy-makers, consumers and their family members, as well as activists, for turning the emerging reality of EBP into a dream which can be meaningfully realized, are huge, and it will be prudent not to expect too much too soon or permit complacence for it not to happen. It is for the service providers and the consumers and their families to work together, to turn the challenge into an opportunity.
